# Antiinflammatory Medicinal Plants from the Ugandan Greater Mpigi Region Act as Potent Inhibitors in the COX-2/PGH_2_ Pathway

**DOI:** 10.3390/plants10020351

**Published:** 2021-02-12

**Authors:** Fabien Schultz, Ogechi Favour Osuji, Barbara Wack, Godwin Anywar, Leif-Alexander Garbe

**Affiliations:** 1 Institute of Biotechnology, Faculty III—Process Sciences, Technical University of Berlin, Gustav-Meyer-Allee 25, 13355 Berlin, Germany; 2 Department of Agriculture and Food Sciences, Neubrandenburg University of Applied Sciences, Brodaer Str. 2, 17033 Neubrandenburg, Germany; favourosuji@yahoo.co.uk (O.F.O.); Barbara.wack@gmail.com (B.W.); garbe@hs-nb.de (L.-A.G.); 3 Department of Plant Sciences, Microbiology and Biotechnology, Makerere University, P.O. Box 7062 Kampala, Uganda; godwinanywar@gmail.com; 4 ZELT—Neubrandenburg Center for Nutrition and Food Technology gGmbH, Seestraße 7A, 17033 Neubrandenburg, Germany

**Keywords:** inflammation, antibiotics, ethnopharmacology, traditional medicine, pain, fever, cyclooxygenase, lipoxygenase, *Albizia coriaria*, *Cassine buchananii*, *Combretum molle*, *Erythrina abyssinica*, *Ficus saussureana*, *Harungana madagascariensis*, *Leucas calostachys*, *Microgramma lycopodioides*, *Morella kandtiana*, *Plectranthus hadiensis*, *Securidaca longipedunculata*, *Sesamum calycinum* subsp. *angustifolium*, *Solanum aculeastrum*, *Toddalia asiatica*, *Warburgia ugandensis*, *Zanthoxylum chalybeum*

## Abstract

Our study investigates 16 medicinal plants via assessment of inhibition of proinflammatory enzymes such as cyclooxygenases (COX). The plants are used by traditional healers in the Greater Mpigi region in Uganda to treat inflammation and related disorders. We present results of diverse in vitro experiments performed with 76 different plant extracts, namely, (1) selective COX-2 and COX-1 inhibitor screening; (2) 15-LOX inhibition screening; (3) antibacterial resazurin assay against multidrug-resistant *Staphylococcus aureus*, *Listeria innocua*, *Listeria monocytogenes*, and *Escherichia coli* K12; (4) DPPH assay for antioxidant activity; and (5) determination of the total phenolic content (TPC). Results showed a high correlation between traditional use and pharmacological activity, e.g., extracts of 15 out of the 16 plant species displayed significant selective COX-2 inhibition activity in the PGH_2_ pathway. The most active COX-2 inhibitors (IC_50_ < 20 µg/mL) were nine extracts from *Leucas calostachys*, *Solanum aculeastrum, Sesamum calycinum* subsp. *angustifolium*, *Plectranthus hadiensis*, *Morella kandtiana*, *Zanthoxylum chalybeum*, and *Warburgia ugandensis*. There was no counteractivity between COX-2 and 15-LOX inhibition in these nine extracts. The ethyl acetate extract of *Leucas calostachys* showed the lowest IC_50_ value with 0.66 µg/mL (COX-2), as well as the most promising selectivity ratio with 0.1 (COX-2/COX-1). The TPCs and the EC_50_ values for DPPH radical scavenging activity showed no correlation with COX-2 inhibitory activity. This led to the assumption that the mechanisms of action are most likely not based on scavenging of reactive oxygen species and antioxidant activities. The diethyl ether extract of *Harungana madagascariensis* stem bark displayed the highest growth inhibition activity against *S. aureus* (MIC value: 13 µg/mL), *L. innocua* (MIC value: 40 µg/mL), and *L. monocytogenes* (MIC value: 150 µg/mL). This study provides further evidence for the therapeutic use of the previously identified plants used medicinally in the Greater Mpigi region.

## 1. Introduction

**Figure 1:**
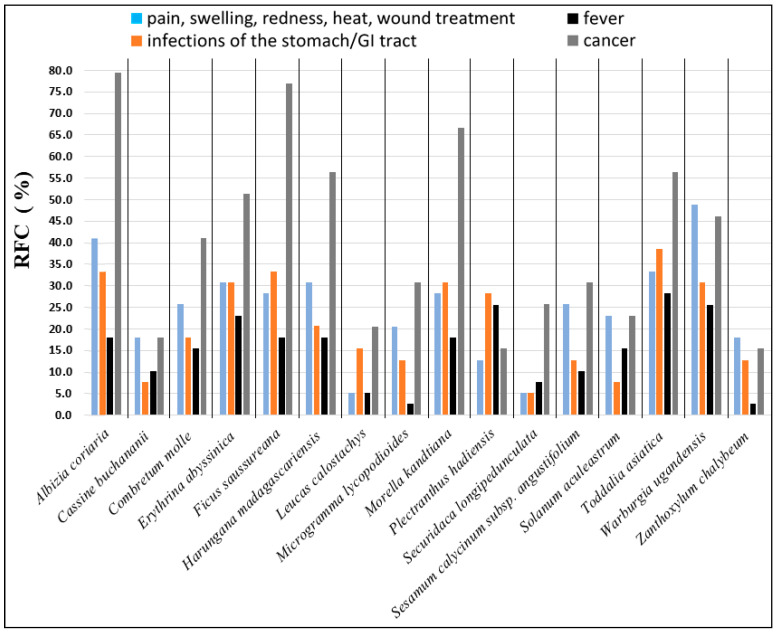
Ethnopharmacological information, describing the traditional use of 16 medicinal plants from the Greater Mpigi region in Uganda (with emphasis on the treatment of cardinal signs of acute inflammation, cancer, and stomach and gastrointestinal (GI) tract infections). The histogram shows the relative frequencies of citation (RFCs) in %, a field assessment index that was calculated from an ethnobotanical survey of 39 traditional healers. The RFC describes the use of plants to treat a specific medical condition relative to the total number of interviewees in the study, assessing the significance of a plant species in the local traditional medicine system (y-axis). This ethnobotanical index can vary from 0% (no survey participant uses this plant in treatment of a specific medical condition) to 100% (all survey participants use this plant in treatment of a specific medical condition) [5].

Approximately 80% of Africa’s population relies almost entirely on plants for medication [1,2,3]. The knowledge of plants in Uganda and their medicinal uses is mainly transferred orally from one generation to the next by traditional healers, leading to the potential for loss of vital information due to lack of records [4,5]. A previous ethnopharmacological study from the Greater Mpigi region documented the traditional use of 39 healers [5]. In this study, Schultz et al. described the medicinal uses of 16 plant species used in treatment of diverse medical disorders. The 16 Ugandan medicinal plant species were *Albizia coriaria*, *Cassine buchananii*, *Combretum molle*, *Erythrina abyssinica*, *Ficus saussureana*, *Harungana madagascariensis*, *Leucas calostachys*, *Microgramma lycopodioides*, *Morella kandtiana*, *Plectranthus hadiensis*, *Securidaca longipedunculata*, *Sesamum calycinum subsp. angustifolium*, *Solanum aculeastrum*, *Toddalia asiatica*, *Warburgia ugandensis*, and *Zanthoxylum chalybeum*. Another study applying the Degrees of Publication (DoP) method as a tool for literature assessment in ethnopharmacological research classified six of these 16 plant species as being “highly understudied” and three species as “understudied” [6]. This DoP analysis further strengthened the justification for conducting pharmacological lab studies, investigating these select medicinal plant species from the Greater Mpigi region. The ethnobotanical survey specifically sought to investigate the treatment of cardinal signs of acute inflammation, which is relevant to the present study. Uses documented for each species include the treatment of pain, fever, redness, heat, wounds, cancer, and general infections [5]. Figure 1 depicts the relative frequencies of citation (RFCs, *n* = 39) for these use reports.

**Figure 2:**
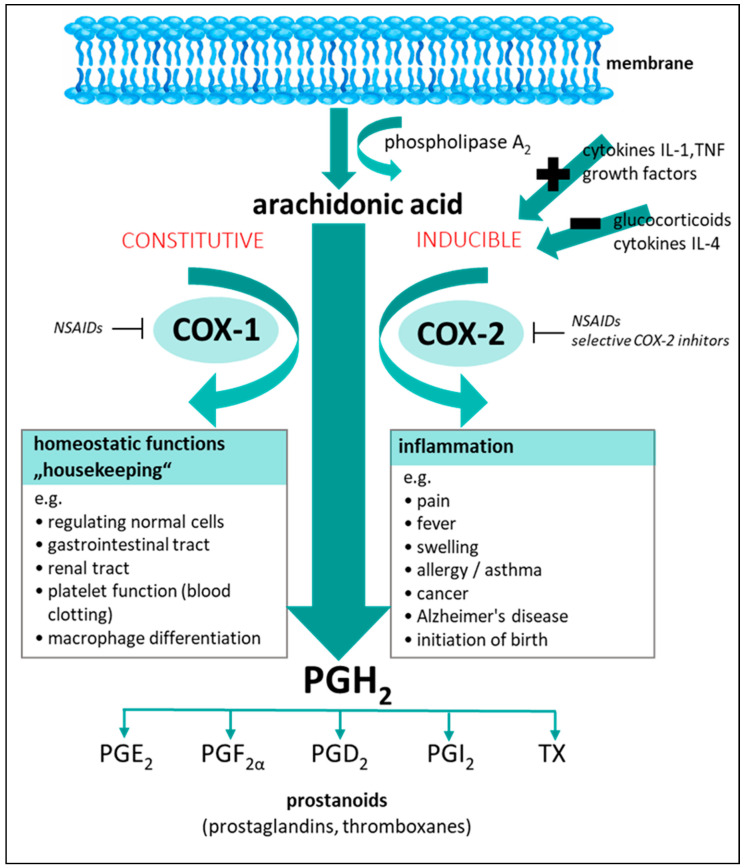
Cyclooxygenase-1/2 pathway and its physiological functions; COX, cyclooxygenase; NSAIDs, nonsteroidal antiinflammatory drugs; IL-1, interleukin 1; IL-4, interleukin 4; TNF, tumor necrosis factor; PGH_2_, prostaglandin H2; PGE_2_, prostaglandin E_2_; PGF_2α_, prostaglandin F_2α_; PGD_2_, prostaglandin D_2_; PGI_2_, prostaglandin I_2_; TX, thromboxane.

Inflammation is the reaction of the immune system to injury and invading pathogens and can be considered one of the most important human host defense mechanisms [5,7,8]. The scientific pursuit of novel antiinflammatory therapeutics and drug leads, e.g., for treatment of pain, is complex and challenging [9,10]. Inflammation has also been implicated in the pathogeneses of diverse medical disorders, and over- or persistent inflammation can cause tissue damage, failure of vital organs, and death [8,11,12]. Its mediators are involved in diverse biochemical signaling pathways. One of these pathways is the cyclooxygenase-1 (COX-1) and cyclooxygenase-2 (COX-2) pathway, which plays a key role in the production of eicosanoids (Figure 2). It is also known as the prostaglandin H_2_ (PGH_2_) pathway, named after the resulting prostaglandin precursor of the COX-catalyzed reaction of arachidonic acid in the human body [7,13]. The main human cyclooxygenases, COX-1 and COX-2, are prostaglandin (PG) endoperoxide synthases (E.C.1.14.99.1) that catalyze the metabolic biosynthesis of arachidonic acid to prostanoids, encompassing potent proinflammatory signaling molecules such as prostaglandin F_2α_ and prostaglandin E_2_ [14,15,16,17]. Each of these COX isoforms catalyzes the reaction of individual prostanoids, whereas products of COX-1 catalysis are involved in normal, homeostatic functions, such as cytoprotection of gastric mucosa, renal blood flow, macrophage differentiation, and hemostasis. These prostaglandins are also involved in regulating normal cells in general, which is why COX-1 is constitutively present in human cells. The concentration in the body generally remains stable [15,18,19,20]. The isoform COX-2, however, plays a major role in inflammatory response. While underexpressed in cells under normal conditions, COX-2 expression is upregulated during inflammation as part of the immune response, rapidly displaying elevated levels. Stimuli that induce COX-2 expression in cells can include proinflammatory cytokines (TNFα, IL-1) or growth factors [8,15,17,18,19,20]. Proinflammatory prostaglandins produced through the COX-2 pathway contribute to or induce pain, fever, and swelling, and are even implicated with types of cancer, allergy, asthma, arthritis, stroke, and Alzheimer’s disease [7,13,19,21,22,23,24,25,26,27,28,29,30,31,32].

Large-scale applied nonsteroidal antiinflammatory drugs (NSAIDs), such as ibuprofen, Paracetamol, or Aspirin, share the capacity for COX/PGH_2_ inhibition, thereby reducing pain, fever, and inflammation. Yet the vast majority of the NSAIDs on the market exhibit no selectivity to COX-1 and COX-2, leading to various side effects caused by inhibition of COX-1 regulated “housekeeping” functions in the body (such as ulceration and gastrointestinal bleeding) [7,20,33,34,35,36,37,38]. In the past, a few selective COX-2 inhibitors were discovered and marketed, e.g., celecoxib and rofecoxib. These drug molecules selectively inhibited COX-2 catalyzed biosynthesis of proinflammatory prostaglandins and were tremendously more potent at inhibiting COX-2 than COX-1 [38,39,40,41]. However, these selective COX-2 inhibitors (members of the diaryl heterocycle group of drug molecules) exhibited severe skin-related and cardiovascular toxicities, including myocardial infarction, leading to partial withdrawal of this class of compounds from the market [8,38,42,43]. As a result, medicinal plants have regained momentum for treatment of inflammatory diseases, making research in this particular field a hot topic [8,17,44,45,46].

**Figure 3:**
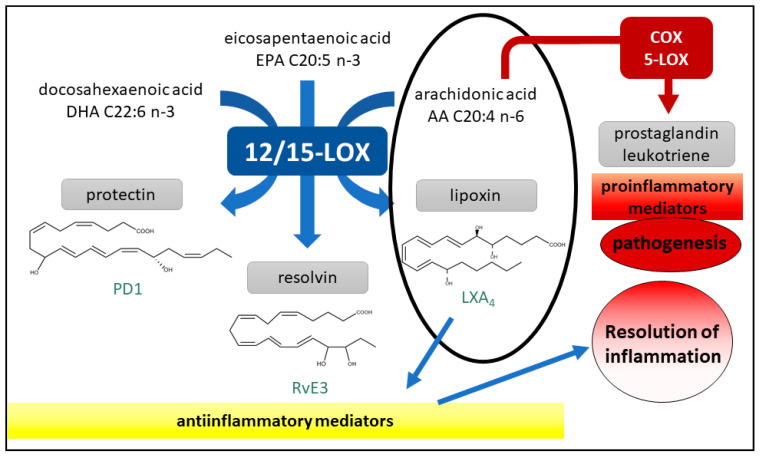
Overview of the 12/15-lipoxygenase (12/15-LOX) pathway, highlighting biosynthesis of the antiinflammatory mediator lipoxin via a 15-*S*-hydroxyeicosatetraenoic acid (15(*S*)-HpETE) precursor with relevance to the 15-LOX inhibition assay presented in this study [48].

Another pathway of inflammatory response in the human body is the lipoxygenase (LOX) pathway. Here, long-chain polyunsaturated fatty acids, such as arachidonic acid, are enzymatically peroxidized by lipoxygenases to the corresponding hydroperoxyl derivatives to produce eicosanoid signaling metabolites (Figure 3) [47].

While COX-2- and 5-LOX-mediated reactions generally produce prostaglandins and leukotrienes that act as proinflammatory mediators involved in pathogenesis, 12/15-LOX generates protectins and resolvins derived from n-3 polyunsaturated fatty acids, as well as lipoxins derived from arachidonic acid (n-6) [48]. A hydroperoxy *cis*-*trans*-1,3-conjugated pentadienyl moiety within the unsaturated fatty acid is the initial, unstable product of the LOX reaction [49,50,51]. Protectins, resolvins, and lipoxins are considered antiinflammatory mediators involved in the regulation of inflammatory responses and resolution of acute inflammation. Therefore, they are required in order to maintain homeostasis [48,52,53]. With regard to lipoxin, there is typically an inverse concentration between antiinflammatory lipoxin and proinflammatory leukotriene present at the site of inflammation [54]. 15-LOX is also involved in the development and progression of cancer, yet its role is complex and still controversial [52]. Due to the presence of two 15-LOX isoforms in human tumor biopsies and its implication in carcinogenesis of some cancers, the literature suggests procarcinogenic as well as anticarcinogenic roles [52,55,56,57,58,59]. Thus, discovery of 15-LOX inhibitors derived from medicinal plants may provide promising, novel, and selective therapies for certain cancers [48,56,57].

We screened up to 76 different extracts derived from these 16 medicinal plants from the Greater Mpigi region in Uganda for antiinflammatory, antioxidant, and antibacterial activity associated with the traditional use of medical disorders described in Figure 1. The main objectives of the study were (a) the pharmacological evaluation of traditional use and (b) contributing to drug discovery. Specifically, the study aims were to investigate the potential in vitro (1) human recombinant COX-2 inhibition activity; (2) human recombinant COX-1 inhibition activity; (3) 15-LOX inhibition activity; (4) free radical scavenging activity; (5) growth inhibitory activity against multidrug-resistant *Listeria innocua*, *Listeria monocytogenes*, *Escherichia coli* K12 and *Staphylococcus aureus*; and (6) to determine the total phenolic content (TPC) of the plant extracts.

## 2. Results

### 2.1. Information on Plant Species and Extractions

Table 1 shows taxonomic information on the 16 medicinal plant species studied, extract identification numbers (extract IDs), extraction solvents used, local names in the Luganda language, plant parts selected for investigation, and herbarium voucher specimen numbers and locations. Extracts were produced through different methods: (a) maceration in either methanol, ethanol, ethyl acetate or diethyl ether, (b) Soxhlet extraction using *n*-hexane and successively methanol, and (c) aqueous decoction, which simulated the original methods of traditional preparation [5].

### 2.2. Selective COX-2 Inhibition Library Screen

**Figure 4:**
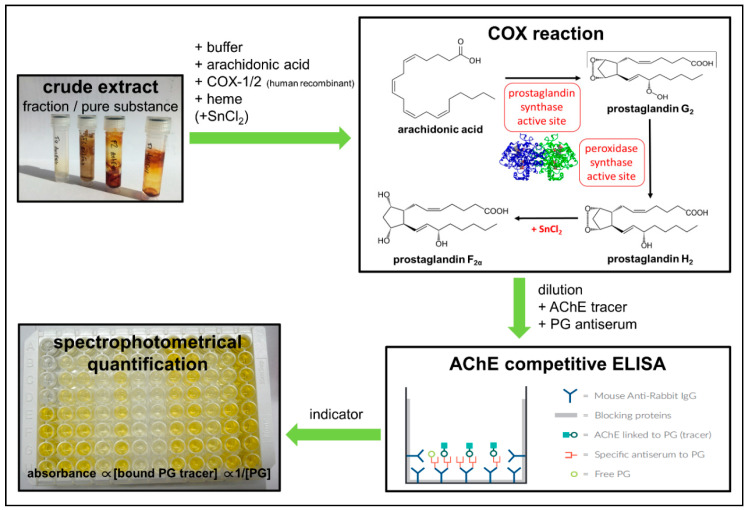
Scheme describing the COX inhibition assay used for screening plant extracts for antiinflammatory activity.

The plant extract library was initially screened for COX-2 inhibition activity at a concentration of 50 µg/mL. Extracts displaying a COX-2 inhibition percentage above 80 were further investigated by dose-response experiments in order to obtain IC_50_ values. The COX (human) inhibition assay has two steps involving a COX reaction and a PG-acetylcholinesterase (AChE) competitive ELISA for direct spectrophotometric quantification of PGF_2α_ by Tin(II) chloride reduction of the PGH_2_ output produced in the COX reaction (Figure 4). The two distinct COX isoforms are bifunctional enzymes, displaying both COX and peroxidase activity [60]. Thus, arachidonic acid is first converted by the prostaglandin synthase active site to a hydroperoxyl endoperoxide (PGG_2_), and then further reduced by the peroxidase synthase active site to the corresponding alcohol (PGH_2_), which is the precursor for PG mediator molecules. The ELISA utilizes a broadly specific antiserum capable of binding to all major PG compounds. It is based on a PG tracer (PG-AChE conjugate) and PGs present in the sample that compete for a limited amount of PG antiserum. Since the PG concentration varies depending on the COX inhibitory activity of plant extracts tested, while the concentration of PG-AChE conjugate is constant, the concentration of PG in the sample is inversely proportional to the amount of PG-AChE conjugate that can bind to the PG antiserum.

In this initial library screen, extracts of 15 out of 16 species inhibited COX-2 at 50 µg/mL. The only exception was extracts of *C. molle*, which did not display any inhibitory activity on COX-2 (I = 0%). Details of the results of the prescreen are given in Supplementary Data Table S1. In total, out of 58 extracts screened, 19 extracts from nine species did not show COX-2 inhibition activity, 15 extracts from 10 species resulted in percentage inhibition of 0–40, and 15 extracts from 10 species exhibited percentage inhibition values between 40 and 80. Nine extracts from seven species were identified as particularly promising due to their high percent inhibition values (%I > 80). These were the ethyl acetate and the *n*-hexane extract of *S. calycinum* subsp. *angustifolium* leaves (eE004, hE004), the ethyl acetate and the *n*-hexane extract of *S. aculeastrum* root (eE006, hE006), the diethyl ether extract of *W. ugandensis* stem bark (dietE014), the ethyl acetate extract of *L. calostachys* leaves (eE005), the diethyl ether extract of *Morella kandtiana* root (dietE012), the diethyl ether extract of *P. hadiensis* leaves (dietE016), and the ethanolic extract of *Z. chalybeum* stem bark (etE009). These nine extracts were selected for the next stage of COX experiments and subsequently introduced to the dose-response COX-2 and COX-1 inhibition studies.

### 2.3. Dose-Response COX-2 Inhibition Experiments

The results of the dose-response COX-2 inhibition experiments, further investigating the most promising nine extracts from seven species identified in the library screen, are reported in Table 2.

Calculated IC_50_ values for these nine extracts ranged from 0.66 to 17.24 μg/mL. The ethyl acetate extract of *L. calostachys* leaves (eE005) displayed the highest inhibitory activity against human recombinant COX-2 in the study (IC_50_: 0.66 μg/mL). The second most active extract in inhibiting COX-2 was the ethyl acetate extract of *S. aculeastrum* root (eE006), reaching an IC_50_ value of 1.74 μg/mL. Further, high COX-2 inhibition activity can be reported for the *n*-hexane extract of *S. aculeastrum* root (hE006; IC_50_: 3.19 μg/mL) and the *n*-hexane extract of *S. calycinum subsp. angustifolium* leaves (hE004; IC_50_: 3.65 μg/mL). There was only one extract among the most active nine extracts that was produced using a polar extraction solvent (ethanol, etE009, *Z. chalybeum* stem bark), meaning that most of the extracts were apolar (extraction solvent: *n*-hexane) or somewhat apolar extracts (extraction solvents: diethyl ether, ethyl acetate).

### 2.4. COX-1 Inhibition Analysis and Selectivity Ratio Determination

The nine most active plant extracts, selected in the initial COX-2 inhibition library screen and followed up on via dose-response COX-2 inhibition studies, were further assayed to assess their potential inhibition activity against human recombinant COX-1. The calculation of the COX-2/COX-1 selectivity ratio for balance of inhibition can be used for the assessment of side effects and efficacy [61,62]. Results are given in Table 2.

All nine extracts inhibited COX-1 enzyme activity and their IC_50_ values ranged from 3.99 to 24.89 μg/mL. Extract eE005, which was previously identified as the strongest COX-2 inhibitor in the extract library, showed a COX-1 inhibition IC_50_ value of 7.76 μg/mL, leading to a calculated COX-2/COX-1 selectivity ratio of 0.1. The second most active COX-2 inhibitor, eE006, displayed moderate COX-1 inhibition activity (IC50: 7.76 μg/mL) and a COX-2/COX-1 selectivity ratio of 0.2. The analysis of extract hE004, which was previously highly active against COX-2, resulted in an IC_50_ value of 8.57 μg/mL and a selectivity ratio of 0.4. The most active COX-1 inhibitors among the nine extracts were hE006 (*n*-hexane extract of *S. aculeastrum* root; IC_50_: 3.99 μg/mL; selectivity ratio: 0.8) and dietE016 (diethyl ether extract of *P. hadiensis* leaves; IC_50_: 5.83 μg/mL; selectivity ratio: 0.8). Two extracts exhibited stronger COX-1 than COX-2 inhibitory effects. These were the diethyl ether extracts of *W. ugandensis* stem bark (dietE014; selectivity ratio: 1.2) and *M. kandtiana* root (dietE012; selectivity ratio: 1.2).

### 2.5. 15-LOX Inhibition Counterscreen

**Figure 5:**
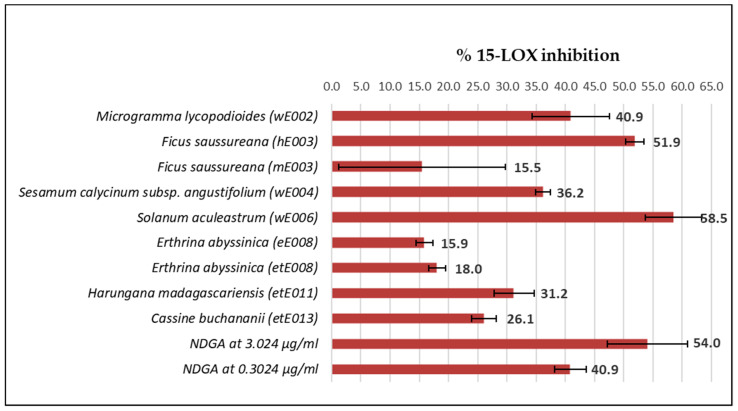
Results of the 15-LOX inhibition extract library counterscreen at 10 μg/mL; positive control tested at 3.024 μg/mL and 0.3024 μg/mL.

In an effort to estimate the 15-LOX counteractivity, the extract library, containing 58 plant extracts previously investigated for COX-2 inhibition activity, was screened at a concentration of 10 μg/mL. The results of extracts inhibiting 15-LOX enzyme activity are reported in Figure 5.

In total, only nine extracts from six plant species exhibited 15-LOX inhibition activity at 10 μg/mL, whereas 49 extracts from 16 species did not display inhibitory activity (I = 0%). These nine extracts were wE002 (aqueous extract, *M. lycopodioides* roots/rhizomes), hE003 and mE003 (*n*-hexane and methanolic extracts, *F. saussureana* stems), wE004 (aqueous extract, *S. calycinum* subsp. *angustifolium* leaves), wE006 (aqueous extract, *S. aculeastrum* roots), eE008 and etE008 (ethyl acetate and ethanolic extracts, *E. abyssinica* stem bark), etE011 (ethanolic extract, *H. madagascariensis* stem bark), and etE013 (ethanolic extract, *C. buchananii* stem bark). Interestingly, except for extracts hE003 and eE008, these active extracts were all polar extracts (extraction solvents: water, methanol, ethanol). Extracts with the highest 15-LOX activity at 10 μg/mL were the aqueous root extract from *S. aculeastrum* (I: 58.5%) and the *n*-hexane stem extract from *F. saussureana* (I: 51.9%).

### 2.6. DPPH Assay for Antioxidant Activity and TPC Determination

**Figure 6:**
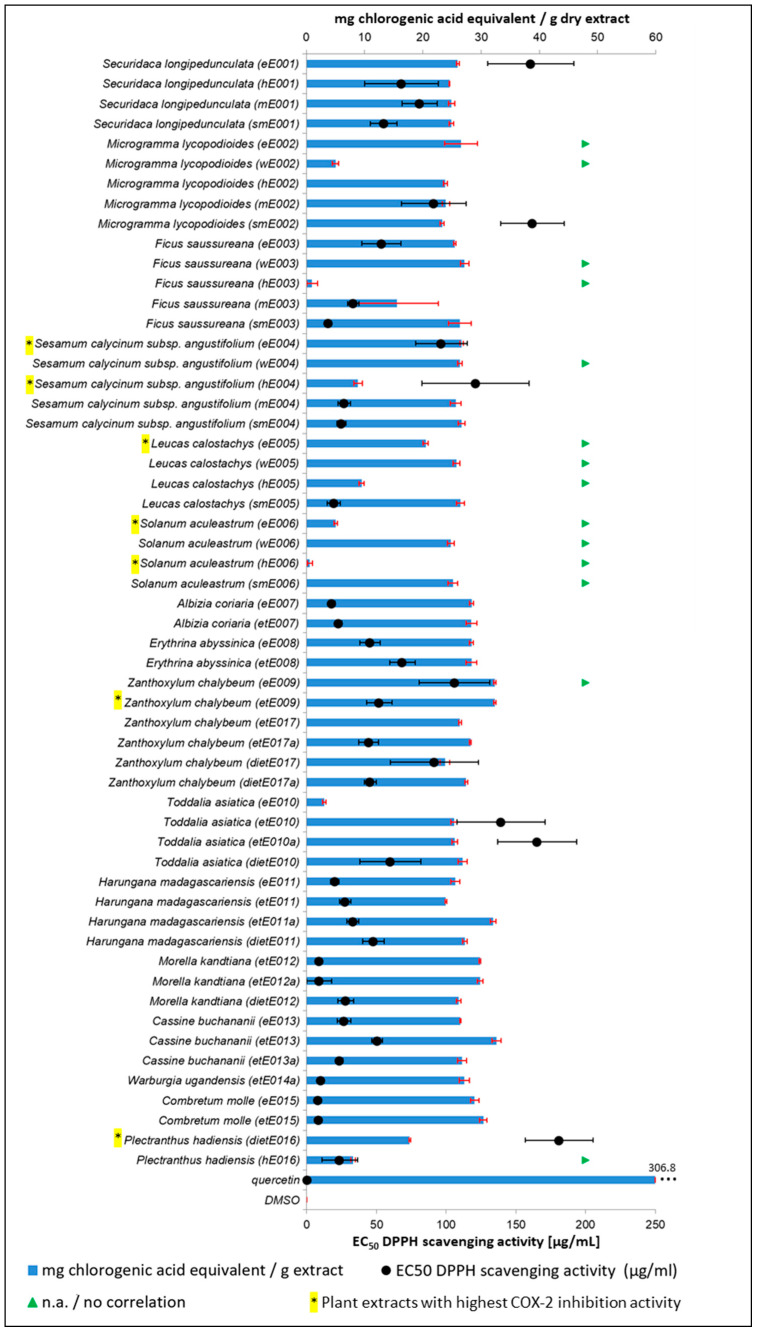
Results of the in vitro investigation of antioxidant activity (free radical scavenging activity) and determination of TPC for assessment of potential mechanism of action of the COX-2/1 and 15-LOX inhibition activity; plant extracts identified in the initial library COX-2 screen for COX-2/COX-1 dose-response inhibition experiments are marked with *; n.a. = not available.

The plant extract library was further screened for free radical scavenging potential (antioxidant activity) and the total phenolic content (TPC) was determined. Both assays were conducted to rule out a potential mechanism of action for the COX-2/1 and 15-LOX inhibition due increased presence of free radical scavenging compounds in highly active plant extracts. Many phenolic compounds, such as tannins or flavonoids, are considered to act via their free radical scavenging activities, facilitating the inhibition of proinflammatory enzymes, e.g., COX and LOX, during host immune response [63,64]. For example, reactivity with the radical trap DPPH (1,1-diphenyl-2-picrylhydrazyl radical) in the presence of each plant extract was evaluated to elucidate the potential of lipid-derived radical scavenging in the mechanism of the 15-LOX enzyme inhibition previously assessed. Results are summarized in Figure 6 and absolute values are reported in Supplementary Table S2.

The results show that there is poor correlation between the TPC and the corresponding EC_50_ values (antioxidant activity), which is further addressed in the Discussion section. Extracts containing the highest TPC in the extract library were etE013 (*C. buchananii;* 32.69 mg chlorogenic acid equivalent/g dry extract), eE009 and etE009 (*Z. chalybeum*; 32.29 mg chlorogenic acid equivalent/g dry extract), and etE011a (*H. madagascariensis*; 32.09 mg chlorogenic acid equivalent/g dry extract). Analysis of hE006 (*S. aculeastrum*; 0.61 mg chlorogenic acid equivalent/g dry extract), hE003 (*F. saussureana*; 1.01 mg chlorogenic acid equivalent/g dry extract), and eE010 (*T. asiatica*; 3.00 mg chlorogenic acid equivalent/g dry extract) resulted in the lowest TPCs in the library.

Interestingly, the two extracts from *C. molle* (the only species inactive in the initial COX-2 inhibition library screen) exhibited the lowest EC_50_ values for free radical scavenging activity in the library, resulting in 8.26 µg/mL (eE015) and 8.73 µg/mL (etE015). Other extracts in a similar EC_50_ range were etE012a and etE012 (*M. kandtiana*; EC_50_: 8.97 µg/mL and 9.03 µg/mL). The highest EC_50_ value for free radical scavenging potential in the extract library was recorded for COX-2 inhibitor dietE016 (*P. hadiensis*; EC_50_: 181.00 µg/mL). A total of 15 extracts did not reach an EC_50_ value in the tested concentration range, which included the top three performing COX-2 inhibitors eE005 (*L. calostachys*), eE006 and hE006 (*S. aculeastrum*), identified above. Except for etE009 (relatively high TPC), none of the nine extracts that exhibited high COX-2 inhibition activity showed an increased antioxidant activity or TPC compared to the other extracts in the library.

### 2.7. Antibacterial Resazurin Bioassay

The plant extract library was further screened for growth inhibition activity against multidrug-resistant strains of *S. aureus* and *E. coli* K12, and *L. innocua* (no resistances reported) to further evaluate their potential for treatment of wounds and infections of the stomach/GI tract. Extracts that were active against the non-pathogenic *L. innocua* were subsequently tested against multidrug-resistant pathogenic *L. monocytogenes*. Minimal inhibition concentration (MIC) values are reported in Table 3.

Compared to the other bacteria tested, the extracts were significantly more active against the Gram-positive *S. aureus* (ATCC 25923). Here, the majority of the plant extracts showed at least a minor inhibitory impact on *S. aureus* growth at the highest concentration tested (500 µg/mL). Thirty-one of 75 plant extracts (41.3%) displayed MIC values below 125 µg/mL. The highest growth inhibitory activity (<50 µg/mL) was recorded for 11 extracts from seven plant species: (1) the methanolic Soxhlet extract of *S. aculeastrum* roots (smE006, MIC: 11.72 µg/mL); (2) the diethyl ether, *n*-hexane, and ethanolic extracts of *H. madagascariensis* stem bark (dietE011, MIC: 13.02 µg/mL; hE011-18 and etE011-18, MIC: 31.25 µg/mL); (3) the methanolic extract of *F. saussureana* stems (mE003, MIC: 20.83 µg/mL); (4) the methanolic Soxhlet extract of *M. lycopodioides* roots/rhizomes (smE002, MIC: 26.04 µg/mL); (5) the *n*-hexane extract of *S. calycinum* subsp. angustifolium leaves (hE004-18, MIC: 31.25 µg/mL); (6) ethyl acetate and ethanolic extracts of *T. asiatica* leaves and stem bark (eE010 and etE010, MIC: 31.25 µg/mL); and (7) the diethyl ether and ethanolic extracts of *W. ugandensis* stem bark (dietE014 and eE014-18: 31.25 µg/mL).

The experiments screening the extract library against multidrug-resistant *E. coli* K12 (ATCC 23716) generally resulted in low growth inhibitory activity. None of the extracts reached a MIC below 250 µg/mL. The antibiotic screen against *L. innocua* (ATCC 33090) resulted in low growth inhibition activities of tested extracts. The exception was extract dietE011 (MIC: 41.67 µg/mL), which is the diethyl ether extract of *H. madagascariensis* stem bark and which was also the second most active extract in the *S. aureus* inhibition bioassays reported above. Seven extracts from four plant species were further investigated for growth inhibition activity against *L. monocytogenes*. The results indicate that extract dietE011 is less effective against this pathogenic strain of *Listeria* than against *L. innocua* (MIC: 125 µg/mL). Extract dietE14 (*W. ugandensis* stem bark extract; also one of the high-performing *S. aureus* growth inhibitors) showed similar activity on *L. monocytogenes*, reaching a MIC of 125 µg/mL.

## 3. Discussion and Conclusions

The results of this study provide scientific evidence for the therapeutic use of medicinal plants from the Ugandan Greater Mpigi region in treatment of inflammatory disorders and infections. Antiinflammatory (COX-2 inhibition) and antibacterial (growth inhibition of *S. aureus*) effects were recorded for most plant species, successfully validating traditional use in 15 out of 16 medicinal plant species investigated in the in vitro studies. The only species exhibiting no COX-2 inhibition activity in the experiments was *C. molle*. All 16 species displayed at least low inhibitory effects on *S. aureus* growth. The determination of the TPC and the assessment of the DPPH radical scavenging activity of the strongest COX-2 inhibitors led to the assumption that a high concentration of phenols and free radical scavenging seem not to play the crucial role in the mechanism of action of the most active plant extracts.

Extracts of the same species distinguished themselves in terms of method of extraction and polarity of extraction solvent used (“pre-fractionation strategy”). The most active COX-2 inhibitors in the extract library were extracts from *L. calostachys* (eE005), *S. aculeastrum* (eE006, hE006), *S. calycinum* subsp. *angustifolium* (eE004, hE004), *P. hadiensis* (dietE016), *M. kandtiana* (dietE012), *Z. chalybeum* (etE009), and *W. ugandensis* (dietE014). There was no counteractivity between COX-2 and 15-LOX inhibition in these nine extracts from seven plant species. Except for the ethanolic extract of *Z. chalybeum* stem bark, all of these highly active extracts were produced using an apolar or somewhat apolar extraction solvent, namely *n*-hexane, ethyl acetate, or diethyl ether. In general, aqueous extracts, whose lab preparation simulated the traditional methods of preparation in the Greater Mpigi region [5], often failed to exhibit bioactive effects in our antiinflammatory and antibacterial in vitro models. The present result is similar to our previous findings that investigated the extract library with other pharmacological test methods [65]. This phenomenon might be explained by the fact that the lab-produced extracts are filtered prior to solvent evaporation as part of extract standardization procedures. This led to the removal of tiny solids present in the traditional preparations, which are normally swallowed by patients along with the infused water. This way, apolar pharmacologically active secondary plant metabolites may remain in the traditional herbal remedy, but only occur in the apolar extracts in our plant extract library.

The ethyl acetate extract of *L. calostachys* leaves (eE005) showed the lowest IC_50_ value for COX-2 inhibition (0.66 µg/mL). With 0.1, this extract also displayed the most promising selectivity ratio (COX-2/COX-1). The IC_50_ value for COX-1 inhibition was 7.76 µg/mL. As a comparison, the IC_50_ values of Aspirin and ibuprofen in the literature are 210 µg/mL and 46 µg/mL (COX-2), and 5 µg/mL and 1 µg/mL (COX-1) [39,40], respectively. This leads to a poor selectivity ratio of 42 (Aspirin) and 46 (ibuprofen), which is characteristic for most commercial NSAIDs [7,36,39,40,41]. Thus, extract eE005 seems to be a much more potent COX-2 inhibitor than Aspirin or ibuprofen, while also displaying much higher selectivity for COX-2 in contrast to COX-1, thereby potentially generating fewer side effects due to decreased COX-1 and increased COX-2 inhibition. The in vitro performance of eE005 can be further highlighted by the fact that it is a crude extract containing a complex mixture of hundreds or thousands of compounds, whereas Aspirin and ibuprofen are pure substances. The TPC content of extract eE005 was not significantly higher than those of the inactive extracts in the library and no EC_50_ value was reached in the DPPH assay at the highest test concentration, indicating low antioxidant activity and eliminating free radical scavenging as a potential mechanism of action. Moreover, extract eE005 only exhibited low growth inhibition activity against *S. aureus* (MIC: 500 µg/mL) and no antibacterial effects on *L. innocua* and *E. coli* (MICs: >500 µg/mL). *L. calostachys* is an aromatic herb occurring in some parts of Uganda. It was recently identified by the DoP method as a “highly understudied” species [6]. In fact, not much research has been done on this species so far. Three studies reported moderate to low antiplasmodial activity of *L. calostachys* crude extracts [66,67,68]. The *n*-hexane extract of the leaves (hE005) displayed significant quorum sensing inhibition activity against the accessory gene regulator (*agr*) system in *S. aureus* [65]. Thus, our study provides the first report of strong in vitro antiinflammatory activity of *L. calostachys*. Other publications in the literature describe the traditional uses of *L. calostachys* in Kenya, which include use for the treatment of ulcers [69,70,71], colic pain in infants, cancer, skin diseases, headache, arthritis, heart diseases [69], malaria [72,73], gastrointestinal disorders [69,71,74,75,76], flu [76,77], and stomach ache [70,76]. Our data provides further evidence for some of these traditional therapeutic uses. According to the authors’ knowledge, there have been no articles published so far reporting isolation and identification of bioactive natural products from *L. calostachys*.

Other strong COX-2 inhibitors identified were the ethyl acetate extract of *S. aculeastrum* root (eE006; COX-2 IC_50_: 1.74 µg/mL; COX-2 IC_50_: 9.72 µg/mL; selectivity ratio: 0.2), the *n*-hexane extract of *S. aculeastrum* root (hE006; COX-2 IC_50_: 3.19 µg/mL; COX-2 IC_50_: 3.99 µg/mL; selectivity ratio: 0.8), and the *n*-hexane extract of *S. calycinum* subsp. *angustifolium leaves* (hE004; COX-2 IC_50_: 3.65 µg/mL; COX-2 IC_50_: 8.57 µg/mL; selectivity ratio: 0.4).

*S. aculeastrum* is a small tree or large shrub with branchlets covered in dense woolly hairs and sharp, curved thorns [6,78]. In our assessment of antioxidant activity, the extracts eE006 and hE006 displayed significantly lower TPCs than other extracts of the same extraction solvent in the extract library (5.06 and 0.61 mg chlorogenic acid equivalent/g extract), as well as no EC_50_ value reached in the DPPH assay. We therefore hypothesize that the mechanism of action for the COX-2 inhibition is not due to free radical scavenging and high phenol content, as often proposed for antiinflammatory medicinal plants [79,80,81,82]. Extract hE006 exhibited moderate antibacterial activity against multidrug-resistant *S. aureus* (MIC: 125 µg/mL) and no inhibitory activity against *L. innocua* (MIC: >500 µg/mL), whereas extract eE006 showed low antibacterial activity against *S. aureus* and *L. innocua* (MIC: 500 µg/mL). Interestingly, a previous study by Schultz et al. [65] identified extract eE006 as one of the two most active extracts in the library for quorum sensing inhibition (*agr* system in *S. aureus*), exhibiting reporter gene subtype-dependent IC_50_ values of 4, 1, 16, and 64 µg/mL (*agr* I-IV). This antivirulence activity was successfully confirmed via a direct protein output assessment (δ-toxin). *S. aculeastrum* is one of the Ugandan species that were recently classified via the DoP method as being “understudied” [6]. Published studies focus on documentation of traditional use, and pharmacological and phytochemical investigation of the berries and the leaves [6], not the roots that were investigated in this study. For instance, these include reports of low antioxidant and antimicrobial activity of the berries and leaves [83,84,85]; antiproliferative activity against human HeLa, MCF7, and HT29 tumor cell lines of methanolic berry extracts [86]; and toxicity studies of berry extracts in Wistar rats [87,88], mainly published by the Afolayan research group at Fort Hare University, South Africa. The new steroidal alkaloids solamargine, β-solamargine, solasodine and tomatidine were isolated from *S. aculeastrum* root bark and berries [89,90,91,92], and solamargine induced P-glycoprotein inhibition and non-selective cytotoxicity [93]. Apart from traditional uses reported from the Greater Mpigi region [5], few other publications also mentioned traditional uses of *S. aculeastrum*, e.g., use of the roots, berries, leaves, and bark to treat cancer in Kenya and South Africa [94,95]; use of the roots to treat stomach ache in South Africa [96]; use of the berry juice to treat ditlapedi (a facial skin condition) in South Africa [97]; and use of the berries and leaves to treat lymphatic filariasis in South Africa [98].

*S. calycinum* subsp. *angustifolium* leaves displayed high COX-2 inhibition activity in this study. This species is an erect, annual to perennial herb with spotted pink or purple flowers, reaching a height of 0.4–2.0 m. It can often be seen in Uganda along the roadside [6,99]. Yet it has also been classified as a “highly understudied” species with regard to ethnopharmacological research [6]. This is because only four publications mention the traditional use of the herb (excluding the study from the Greater Mpigi region [5]) [6]. To briefly summarize, *S. calycinum* subsp. *angustifolium* is used in the treatment of burns, wounds, eye infections, and diarrhea, and as a contraceptive and emetic in Tanzania [100]. In Uganda, it was reported to be used to treat hernias [101], to induce vomiting [102], and to treat hypertension in combination with other herbs [103]. There have been no pharmacological investigations published on this species so far, except for the antibiotic, cytotoxicity, and antivirulence study investigating the same extract library [65]. Here, extract hE004, just as extract eE006, was among the two most active *agr* system quorum sensing inhibitors (IC_50_ values: 2, 2, 16, and 32 µg/mL (*agr* I-IV)). The antiinflammatory activity of the *n*-hexane extract of *S. calycinum* subsp. *angustifolium* leaves (hE004) described in this study is the first report in the literature to date, as well as the first scientific evidence for its therapeutic use in the Greater Mpigi region of Uganda.

Phenolic compounds are often thought to possess antiinflammatory properties. The mechanisms of action of many phenolic compounds are most likely associated with their inhibition of proinflammatory enzymes in the arachidonic acid pathway (e.g., COX-2 and 5-LOX) or with their free radical scavenging activity [79,80,81,82]. Except for extract etE009 (relatively high TPC), none of the nine extracts that exhibited high COX-2 inhibition activity showed an increased antioxidant activity or calculated TPC compared to the other extracts in the library. Interestingly, the lowest EC_50_ value for free radical scavenging/antioxidant activity was recorded for the two extracts of *C. molle* (eE015, EC_50_: 8.26 µg/mL; etE015, EC_50_: 8.73 µg/mL), the only species that did not display any inhibitory activity on COX-2 in the initial library screen. These findings suggest that free radical scavenging and a high concentration of phenols in general seem not to be involved in the mechanism of action of the most active COX-2 inhibiting plant extracts. In theory, there is a direct relationship between the TPC and the free radical scavenging activity because phenols significantly contribute to the antiradical activity [104]. The poor correlation between high TPC and low EC_50_ values reported for some samples may be attributed to the different quality of phenols present in the samples, resulting in varying antioxidant activities. Constituents other than phenols, such as carbohydrates, fatty acids, phospholipids, etc., may also play a role in the antioxidant activity of the sample. Correlations between the phytochemical composition of the plant species and their bioactive properties should be further investigated by more advanced methods of analytical chemistry, such as LC-MS-MS profiling, enabling identification of specific sets of molecules present in the extract.

In the antibiotic resazurin bioassay, the diethyl ether extract of *H. madagascariensis* stem bark (dietE11) displayed high growth inhibition activity against *S. aureus* (MIC value: 13 µg/mL), *L. innocua* (MIC value: 42 µg/mL), and *L. monocytogenes* (MIC value: 125 µg/mL). *H. madagascariensis*, the “orange-milk tree,” is an evergreen shrub or tree whose sap is orange and turns blood-red upon exposure [105,106,107]. It is not considered an understudied species because it has been extensively studied in the past [6]. Traditional use has been reported in many regions of the African continent [73,108,109,110,111,112].

Another plant extract exhibiting strong growth inhibitory effects against *S. aureus* was the diethyl ether extract of *Z. chalybeum* stem bark (dietE017a; MIC: 13 µg/mL). This species is a spiny deciduous tree or shrub, reaching heights of about 8 m. It occurs in dry woodland, bushland, or grassland in medium to low altitudes throughout Uganda (up to 1500 m.a.s.l.) [105,106,107]. According to the DoP analysis [6], *Z. chalybeum* is regarded a “moderately studied species.” None of the traditional healers were cited to use this medicinal plant as a remedy for skin infections. However, 18% of the survey participants stated that it is used for disinfection of wounds, as well as treatment of sore throat (8%) and disorders of the stomach/GI tract (13%) (Figure 1 and [5,65]). The present data, therefore, supports the traditional use of *Z. chalybeum* stem bark in the study area.

A polar extract of *S. aculeastrum* root (smE006; methanolic Soxhlet extraction) displayed the highest antibacterial activity against *S. aureus* with a MIC of 12 µg/mL (species discussed above). The results of the antibiotic resazurin assay against multidrug-resistant *S. aureus* strongly support the traditional medicinal use of *S. aculeastrum*, *H. madagascariensis*, and *Z. chalybeum* in treatment of wounds and infections.

Another bioactive medicinal plant that inhibited the growth of *S. aureus*, *L. innocua*, and *L. monocytogenes* is *S. longipedunculata*. This species is mentioned in the literature in connection with treatment of measles [113]. In Uganda, measles is one of the major diseases responsible for fatalities in children. Pneumonia is the most common severe complication, leading to the most measles-associated deaths. It can be caused by the measles virus alone, secondary viral infection, or also secondary bacterial infections. Here, *S. aureus* is the most abundant organism of secondary bacterial infections [114]. In parts of Uganda, the roots of *S. longipedunculata* are prescribed by traditional healers to control secondary bacterial and measles infections [115].

The same plant extract library was previously screened in another study against a panel of multidrug-resistant ESKAPE pathogens, in which we used a different method to test antibacterial growth inhibition [65]. The bacterial species and strains tested also varied, however, the *S. aureus* (UAMS-1 strain) can be compared to the *S. aureus* strain (ATCC 25923) used in this study. Generally, some MIC values differed. However, many values were confirmed by the resazurin bioassay in our study, e.g.:

- extract hE005-18 (*L. calostachys*, MIC_UAMS-1_: >256 µg/mL, MIC_25923_: 500 µg/mL);
- extract hE006 (*S. aculeastrum*, MIC_UAMS-1_: 128 µg/mL, MIC_25923_: 125 µg/mL);
- extract etE008 (*E. abyssinica*, MIC_UAMS-1_: 64 µg/mL, MIC_25923_: 63 µg/mL);
- extract etE011-18 (*H. madagascariensis*, MIC_UAMS-1_: 32 µg/mL, MIC_25923_: 31 µg/mL);
- extract hE011-18 (*H. madagascariensis*, MIC_UAMS-1_: 32 µg/mL, MIC_25923_: 31 µg/mL); and
- extract etE013 (*C. buchananii*, MIC_UAMS-1_: >256 µg/mL, MIC_25923_: 500 µg/mL).

For some extracts, approximately one additional two-fold dilution could be reported as MIC value against *S. aureus*:

- extract dietE011 (*H. madagascariensis*, MIC_UAMS-1_: 32 µg/mL, MIC_25923_: 13 µg/mL);
- extract dietE017a (*Z. chalybeum*, MIC_UAMS-1_: 32 µg/mL, MIC_25923_: 13 µg/mL);
- extract dietE014-18 (*W. ugandensis*, MIC_UAMS-1_: 64 µg/mL, MIC_25923_: 31 µg/mL); and
- extract hE014-18 (*W. ugandensis*, MIC_UAMS-1_: 64 µg/mL, MIC_25923_: 31 µg/mL).

Extract smE006, which was the strongest *S. aureus* growth inhibitor in this study, did not display antibiotic effects against the UAMS-1 strain [65]. A similar result was obtained for extract dietE10 (*T. asiatica*). This significant discrepancy can most likely be explained by the different resistance profiles of the two strains investigated. The results of this study also showed that the antibacterial potential of medicinal plants depends on the species, which plant part is used, the time and location of harvest, and on the solvents used for extraction.

The results of this study, reporting pharmacological effects of medicinal plants on inflammatory enzyme cascades and growth of bacterial pathogens, could be the starting point for subsequent studies to investigate potential leads for the development of potent antiinflammatory drugs or antibiotics. Further work is required to characterize the extracts phytochemically in order to identify compounds responsible for the antiinflammatory, antioxidant, and antibacterial properties of the plant species. This could be achieved, for instance, via bioassay-guided fractionation experiments and investigation of the mechanisms of action. However, these effects might also be due synergistic relationships of multiple active ingredients within the plant extracts. To assess the plant species’ potential for drug discovery endeavors, future research should also include evaluation of toxicity (e.g., cytotoxicity and genotoxicity) and in vivo studies. Regarding future in vivo studies and for more accurate validation of traditional use, it will also be essential to continue including the original preparation cited by the traditional healers in the experimental setup, as well as their route of administration and dose. Extracts that displayed strong antibacterial activity need to be further investigated regarding their ability to limit the severity of disease, as well as their potential of increasing the efficacy of conventional antibiotics (that pathogens may have acquired resistance to already). Future studies should therefore also focus on the deactivation of other virulence pathways, such as secretion systems and biofilm formation.

## 4. Materials and Methods

### 4.1. Ethnobotanical Data

Ethnobotanical information on the traditional use of the 16 plant species for treatment of inflammatory disorders was obtained from a previously published survey among 39 traditional healers in the Greater Mpigi region in Uganda [5]. Traditional use reports from this study served as the basis for antiinflammatory, antioxidant, and antibacterial experiments.

### 4.2. Collection and Identification of Plant Material

Following standard collection procedures [116,117,118] and under guidance of the traditional healers, plant specimens were collected during fieldwork in 2015, 2016, and 2017. For all collected samples of species, voucher specimens were prepared and deposited at the Makerere University Herbarium in Kampala, Uganda. Additional select specimens were deposited at the Emory University Herbarium (GEO) in Atlanta, GA, USA, which are also digitally available on the SERNEC portal [119]. Voucher specimen numbers are given in the Table 1. Plant identification and assignment of scientific names (cross-checked with http://www.theplantlist.org) (accessed on 11 August 2019) were conducted following the best practice in the field of ethnopharmacology [120]. Assignments of plant family correspond to The Angiosperm Phylogeny Group IV guidance [121].

### 4.3. Extractions

Collected plant samples were dried in the shade, taken to the laboratory, and ground. Extractions were performed as described in detail in the flow sheet of the Supplementary Material Figure S2 of a previous publication [65]. Briefly, methods applied to extract plant samples were either maceration, aqueous decoction, or Soxhlet extraction. Extraction procedures were conducted using different solvents, aiming to achieve selective extraction of biomolecules of different polarities from the samples. Individual crude extract samples were labeled according to their extraction solvent and the collection number (EXXX) assigned to a plant species during the field studies, ranging from E001 to E017. Regarding the maceration procedure, the extraction solvents used were (a) methanol (mEXXX), (b) ethanol (etEXXX), (c) ethyl acetate (eEXXX), and (d) diethyl ether (dietEXXX). According to the ethnobotanical survey [5], traditional healers usually prepare their herbal drugs as aqueous decoctions. In order to simulate this original method of preparation, plant samples were also boiled in (e) water at 95 °C for 30 min while being stirred (wEXXX). Soxhlet extraction crude extracts were extracted using (f) *n*-hexane (hEXXX) or (g) methanol (smEXXX; successive extraction of corresponding EXXX material). Some of the plant species had to be collected again in 2018 due to the requirement to obtain higher amounts of extract, e.g., for future bioassay-guided fractionation strategies. Their resulting extracts were additionally labeled with “−18” in their sample ID.

### 4.4. Sample Preparation

Crude extracts were dissolved in DMSO (Carl Roth) at 10 mg/mL. Sonication and temperature increase up to 55 °C were applied for some samples with moderate or low solubility experienced at RT. Extract solutions were stored at −20 °C until assaying.

### 4.5. COX-1/2 Inhibition Screening Assays

First, an initial COX-2 inhibition library screen was performed at 50 μg/mL. Extracts exhibiting a COX-2 inhibition value above 80% were introduced to the COX-2 and COX-1 dose-response studies.

Materials and chemicals for the COX inhibition screening assays were sourced from Cayman Chemical, Ann Arbor, MI, USA (Cayman Item No. 701070-96 and 701080-96). This assay for assessment of human recombinant cyclooxygenase inhibitory potential of plant extracts was divided into two steps (described in the following paragraphs): (1) the COX reaction step and (2) the ELISA step. The ELISA step was performed to quantify the prostaglandin product generated in the COX reaction step.

COX reaction step: 5 mL reaction buffer (Cayman Item No. 460104) was mixed with 45 mL ultrapure water (COX buffer). 80 μL human recombinant COX-2 (Cayman Item No. 460121) or COX-1 (Cayman Item No. 460108) solution were diluted with 320 μL of COX buffer and stored on ice, resulting in a quantity sufficient for 40 COX reactions (COX solution). To obtain a heme solution necessary for the reaction, a commercial heme in DMSO solution (Cayman Item No. 460102) was further diluted (40 μL with 960 μL of COX buffer; stable at room temperature for 12 h). 50 μL of arachidonic acid in ethanol (Cayman Item No. 460103) were mixed with 50 μL of 0.1 M potassium hydroxide, vortexed, and further diluted with 400 μL of ultrapure water (final concentration of substrate solution: 2 mM; to be used within 1 hour if kept on ice). 5 mL of 1 M hydrochloric acid were added to a crystalline stannous chloride vial (Cayman Item No. 460107) and vortexed to obtain a saturated solution, which was stable for 8 hours at RT. The selective COX-2 inhibitor compound DuP-769 was used as a positive control. The COX reaction procedure is described in Supplementary Table S4.

Briefly, the background tubes were used to generate the background values. These two tubes contained an inactivated COX solution (10 μL; produced by placing a 500 μL microfuge tube containing 20 μL COX solution in boiling water for 3 min), 160 μL of COX buffer, and 10 μL of heme solution. The sample (or positive control) tubes and two COX 100% initial activity tubes were prepared by adding 160 μL of COX buffer, 10 μL of heme solution, and 10 μL COX solution. 10 μL of DMSO (sample vehicle) were added to each background and COX 100% tube. 10 μL of sample or positive control solution were then transferred to each sample tube. All tubes were incubated for 10 min at 37 °C. Initiation of the COX reaction was completed by adding 10 μL of arachidonic acid substrate solution and incubating for exactly 2.00 min at 37 °C (final substrate concentration in the reaction: 100 μM). The enzymatic reaction was stopped through the addition of 30 μL of saturated stannous chloride solution and the tubes were further incubated for 5 min at RT. The tubes were then tightly capped and stored at 4 °C for up to 3 days (produced PG F_2α_ is stable for one week). A more detailed description of the assay procedure is available [122].

ELISA step: PG F_2α_ was then quantified via Cayman Chemical’s Prostaglandin Screening AChE ELISA kit (Cayman Item No. 514012). The AChE competitive ELISA procedure was performed according to the manufacturer’s instructions [122,123]. Briefly, the COX reaction tubes, containing prostanoids, were diluted in ELISA assay buffer (1:2000 and 1:400 dilutions were run in the ELISA). A prostaglandin standard screening solution was freshly prepared and diluted (twofold), ranging from 2000.0–15.6 pg/mL. Each plate setup contained a minimum of two blanks, two non-specific binding wells, two maximum binding wells, one total activity well, an eight-point standard curve run in duplicate, and the COX 100% activity and COX reaction samples at 1:2000 and 1:4000 dilution in duplicate, respectively. Samples and controls were transferred to pre-coated mouse monoclonal anti-rabbit IgG antibodies. After the addition of a PG-AChE tracer to each well (except for the total activity and the blank wells) and a specific PG antiserum, the plate was incubated for 18 hours at room temperature on a rotary microtiter plate shaker. After incubation, the wells were emptied and rinsed five times with wash buffer in order to remove all unbound reagents. The plate was developed by adding Ellmann’s reagent (AChE substrate) to all wells, as well as addition of the tracer to the total activity wells, and shaking on a rotary microtiter plate shaker in the dark for 75 min. The yellow color of the reaction product of AChE was then measured spectrophotometrically at 412 nm. The intensity is proportional to the amount of PG tracer bound to the well (determined from the PG standard curve from the plate), thus inversely proportional to the quantity of free PG present in the well (Figure 4).

The calculation of % COX inhibition values was performed using the Cayman Chemical’s Workshop Sheets Excel template (Version 11 October 2011). IC_50_ values were calculated using the GraphPad Prism 9.0.0 software and a log(inhibitor) vs. response—variable slope (four parameters) model with duplicate and triplicate determinations. Error propagation was performed as described by the software manufacturer [124] and in the literature [125].

### 4.6. 15-LOX Inhibition Assay

**Figure 7:**
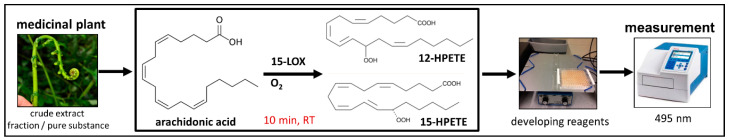
LOX-15 inhibition assay flow sheet depicting the experimental procedure.

The extract library previously screened for COX-2 inhibition activity was subsequently counterscreened for 15-LOX inhibition activity at 10 μg/mL. The 15-LOX inhibition assay was performed using a lipoxygenase inhibitor screening assay kit, manufactured by Cayman Chemical, Ann Arbor, MI, USA (Cayman Item No. 760700). This 96-well microtiter plate-based method utilizes the 15-LOX catalyzed enzymatic reaction between a polyunsaturated free fatty acids with a *cis,cis*-1,4-pentadiene-type structure and molecular oxygen (Figure 7). In the assay, the hydroperoxides, namely 12(*S*)-hydroxyeicosatetraenoic acid (12(*S*)-HpETE) and 15(*S*)-hydroxyeicosatetraenoic acid (15(*S*)-HpETE), are detected and measured.

Purified soybean 15-LOX (Cayman Item No. 760714) was used to facilitate lipoxygenation (final concentration in each well: 200 U/mL). Arachidonic acid was selected as the substrate (final concentration in each well: 125 mM). Nordihydroguaiaretic acid (NDGA), a non-selective LOX inhibitor, was used as a positive control (Cayman Item No. 760717). The assay was performed according to the manufacturer’s instructions [126] and as previously described [127,128,129,130]. Briefly, individual plant extract solutions were incubated with LOX assay buffer containing 15-LOX for 5 min at RT. Three blank control wells, three 100% initial activity wells, three negative control wells, and NDGA positive control wells were also included in each plate setup. After the addition of the substrate and initiation of the reaction, the uncovered plate was placed on a shaker at 500 rpm for 20 min. After incubation, chromogen was rapidly added to stop enzyme catalysis and the plates were covered and placed on a shaker at 500 rpm for 5 min to develop the reaction. The absorbance at 495 nm was then read using a plate reader.

All experiments were conducted in triplicate. During data analysis, the average absorbance of the blank, 100% initial activity, positive control, and sample wells were determined. After subtraction of the average blank absorbance from the average 100% initial activity and sample wells, the % inhibition for each sample was calculated using the following equation:

% inhibition = ((100% initial activity − sample)/100% initial activity) * 100

### 4.7. DPPH Assay

A quantitative DPPH assay for free radical scavenging activity (antioxidant potential) of plant extracts was conducted as previously described [131,132,133]. The 96-well microtiter plates were incubated in the dark for 30 min at RT. Absorbance was measured at 517 nm via UV-vis spectrophotometer. All plant extracts were assayed at 500 µg/mL in their initial well and double-fold diluted down to 0.24 µg/mL. Quercetin was used as a positive control and DMSO as the vehicle control. All experiments were performed in triplicate. The % inhibition was calculated with the following equation:

% inhibition = ((absorbance_blank_ − absorbance_sample_)/absorbance_blank_) * 100

The EC_50_ values were calculated via linear regression using Microsoft Excel^®^.

### 4.8. TPC Determination

Determination of the total phenolic content (TPC) of plant extracts was performed in 96-well microtiter plates as previously described [134]. Briefly, Folin–Ciocalteu reagent was used and a standard curve with chlorogenic acid (CHA) was prepared (serial dilution ranging from 100 µg/mL (or 282.24 µM) to 0.049 µg/mL (or 0.1383 µM)). Spectrophotometric measurement was conducted at 765 nm. Quercetin was used as a positive control and DMSO as a vehicle control. All experiments were performed in triplicate. For data analysis, the CHA standard curve was plotted and linear regression applied using the software GraphPad Prism 9.0.0. After subtracting plant extract blank absorbances from the sample absorbance, the results were interpolated in the standard curve to determine the equivalent CHA concentration for each extract.

### 4.9. Bacterial Strains

In order to realistically evaluate the extracts’ potential for future drug discovery advances for antimicrobial resistance threats, multidrug-resistant isolates of three bacterial species, (a) *E. coli* K12 (ATCC 23716), (b) *S. aureus (ATCC 25923)*, and (c) *L. monocytogenes (ATCC 15313),* were selected for experiments. As a prescreen, a non-pathogenic *L. innocua* strain (ATCC 33090) was used as a substitute (no resistances reported), and hits were followed up with the human pathogen *L. monocytogenes*. Antibiotic resistance profiles, strain numbers and characteristics, and sources are reported in Supplementary Table S3. All strains were streaked from freezer stock and maintained on tryptic soy agar (TSA) via overnight incubation at 37 °C. Overnight liquid cultures were prepared in Mueller-Hinton (MHB, *S. aureus*, *E. coli*) or brain heart infusion (BHI) broth at 37 °C with constant shaking at 200 rpm. For *S. aureus*, a bacterial growth curve was generated, allowing for determination of the growth phase and CFU/mL for standardization. After incubation of the overnight culture, 1.0 mL of the bacteria culture was taken and pipetted into a sterile flask containing 30 mL of broth. After 5–6 h of incubation, the bacteria were in the exponential phase according to the growth curve. In order to get rid of the preculture broth medium, 20 mL of the bacterial culture were centrifuged at 4000 rpm for 5 min. The supernatant was discarded, and the bacterial pellet was resuspended in 20 mL of sterile saline. After vortexing, the suspension was centrifuged again under the same conditions as stated above. Those steps were repeated three times until the supernatant was clear. The pellet was once again resuspended in 20 mL sterile saline and the optical density (OD) was measured at 600 nm. The OD of the culture and the growth curve were used to calculate the dilution factor to achieve a final concentration of 5 × 10^6^ CFU/mL for the resazurin bioassay. As a control and to ensure correct bacterial concentration in the assay, the bacterial suspension was additionally spread onto TSA plates, incubated overnight at 37 °C, and colonies were counted. The other three strains were standardized via cell counting in a Thoma chamber, followed by calculation of the cell concentration, calculation of the correct dilution factor, and dilution.

### 4.10. Resazurin Bioassay

An in vitro 96-well microtiter plate-based antibacterial bioassay, incorporating resazurin as a colorimetric indicator of cell growth, was used to assess the growth inhibitory effects of plant extracts against tested bacterial pathogens. The method was previously described by Sarker et al. [131,135]. The plates were labelled as shown in Supplementary Material Figure S1. Sterility and vehicle/growth controls, as well as a positive control (ciprofloxacin), were incorporated on each microtiter plate of the bioassay. A schematic description of the bioassay procedure is given in Supplementary Material Figure S2. Briefly, 50 µL of sterile saline was pipetted in all wells, except for the first row (20 µL of sterile saline). Next, (a) in column 1 to 4 80 µL of extract solution, (b) in the two X columns 80 µL of sterile saline (sterility control), (c) in column Y 80 µL of DMSO (vehicle control), and (d) in column Z 80 µL of ciprofloxacin (10 µg/mL) as a positive control were added. A serial dilution from row 1 to row 12 was performed. After each dilution step, the pipette tips were discarded. A total of 30 µL of 3.3 x strength MHB and 10 µL of resazurin solution (0.05% (*w/v*) resazurin sodium salt in sterile ultrapure water) were added to all wells. Except for column X, all wells were inoculated with 10 µL of standardized bacterial suspension, resulting in a final bacterial concentration of 5 × 10^5^ CFU/mL in the wells. An amount of 10 µL of sterile saline was added to the sterility control (column X). The plate was sealed with a microtiter plate foil to prevent draining and the plate was shaken on an orbital shaker at 500 rpm for 5 min. The plate was then incubated in the dark for 18 h at 37 °C. After incubation, the MIC was determined by visual assessment of the color change. Any color change from blue to pink was recorded as negative, indicating bacterial growth. Blue was interpreted as inhibition of growth by the individual plant extract (Supplementary Material Figure S1). The lowest concentration at which color change occurred was taken as the MIC value. All bacterial experiments were performed as biological triplicates.

## Figures and Tables

**Table 1:** Description of collected plant species and different extracts investigated in this study.

Scientific Name	Family	Local Name in Luganda	Plant Part	Voucher Specimen Number and Location	Extraction Solvent	Extract ID
*Securidaca longipedunculata* Fresen.	Polygalaceae	Mukondwe	stem	AG196 (Makerere University herbarium, Uganda)	ethyl acetate ^1^	eE001
					water ^1^	wE001
					*n*-hexane (sox.) ^1^	hE001
					methanol ^1^	mE001
					methanol (sox. succ.) ^1^	smE001
*Microgramma lycopodioides* (L.) Copel.	Polypodiaceae	Kukumba	root (rhizomes)	AG639 (Makerere University herbarium, Uganda)	ethyl acetate ^1^	eE002
					aqueous ^1^	wE002
					*n*-hexane (sox.) ^1^	hE002
					methanol ^1^	mE002
					methanol ^1^ (sox. succ.)	smE002
*Ficus saussureana* DC.	Moraceae	Muwo	stem	AG219 (Makerere University herbarium, Uganda)	ethyl acetate ^1^	eE003
					aqueous ^1^	wE003
					*n*-hexane (sox.) ^1^	hE003
					methanol ^1^	mE003
					methanol (sox. succ.) ^1^	smE003
*Sesamum calycinum* subsp. *angustifolium* (Oliv.) Ihlenf. & Seidenst.	Pedaliaceae	Lutungotungo	leaves	AG205 (Makerere University herbarium, Uganda) 23173 * (Emory University herbarium, USA)	ethyl acetate^1^	eE004
					water ^1^	wE004
					*n*-hexane (sox.) ^1^	hE004
					methanol ^1^	mE004
					methanol (sox. succ.) ^1^	smE004
					ethyl acetate^5^	eE004-18
					*n*-hexane (sox.) ^5^	hE004-18
*Leucas calostachys* Oliv.	Lamiaceae	Kakuba musulo	leaves	AG195 (Makerere University herbarium, Uganda) 23175 * (Emory University herbarium, USA)	ethyl acetate ^1^	eE005
					water ^1^	wE005
					*n*-hexane (sox.)^1^	hE005
					methanol (sox. succ.) ^1^	smE005
					ethyl acetate ^5^	eE005-18
					*n*-hexane (sox.) ^5^	hE005-18
					methanol ^5^	mE005-18
					methanol (sox. succ.) ^5^	smE005-18
*Solanum aculeastrum* Dunal	Solanaceae	Kitengo	root	AG193 (Makerere University herbarium, Uganda)	ethyl acetate ^1^	eE006
					water ^1^	wE006
					*n*-hexane (sox.) ^1^	hE006
					methanol (sox. succ.) ^1^	smE006
*Albizia coriaria* Oliv.	Fabaceae	Mugavu	stem bark	AG203 (Makerere University herbarium, Uganda)	ethyl acetate ^2^	eE007
					ethanol ^2^	etE007
*Erythrina abyssinica* DC.	Fabaceae	Jjirikiti	stem bark	AG199 (Makerere University herbarium, Uganda)	ethyl acetate ^2^	eE008
					ethanol ^2^	etE008
*Zanthoxylum chalybeum* Engl.	Rutaceae	Ntaleyaddungu	stem bark	AG204 (Makerere University herbarium, Uganda)	ethyl acetate ^2^	eE009
					ethanol ^2^	etE009
					ethanol ^3^	etE017
					diethyl ether ^3^	dietE017
					ethanol ^4^	etE017a
					diethyl ether ^4^	dietE017a
*Toddalia asiatica* (L.) Lam.	Rutaceae	Kawule	leaves, bark	AG190 (Makerere University herbarium, Uganda)	ethyl acetate ^2^	eE010
					ethanol^2^	etE010
					diethyl ether ^4^	dietE010
					ethanol ^4^	etE010a
*Harungana madagascariensis* Lam. ex Poir.	Hypericaceae	Mukabiiransiko	stem bark	AG230 (Makerere University herbarium, Uganda) 23180 * (Emory University herbarium, USA)	ethyl acetate ^2^	eE011
					ethanol ^2^	etE011
					diethyl ether ^4^	dietE011
					ethanol ^4^	etE011a
					ethyl acetate ^5^	eE011-18
					*n*-hexane ^5^	hE011-18
					ethanol ^5^	etE011-18
					diethyl ether ^5^	dietE011-18
*Morella kandtiana* (Engl.) Verdc. & Polhill	Myricaeae	Mukikimbo	root	AG201 (Makerere University herbarium, Uganda) 23174 * (Emory University herbarium, USA)	ethyl acetate ^2^	eE012
					ethanol ^2^	etE012
					ethanol ^4^	etE012a
					diethyl ether ^4^	dietE012
					ethyl acetate ^5^	eE012-18
					diethyl ether ^5^	dietE012-18
*Cassine buchananii* Loes.	Celastraceae	Mbaluka	stem bark	AG198 (Makerere University herbarium, Uganda)	ethyl acetate ^2^	eE013
					ethanol ^2^	etE013
					ethanol ^4^	etE013a
*Warburgia ugandensis* Sprague	Canellaceae	Abasi	stem bark	AG220 (Makerere University herbarium, Uganda) 23181 * (Emory University herbarium, USA)	ethanol ^4^	etE014a
					diethyl ether ^4^	dietE014
					ethyl acetate ^5^	eE014-18
					water	wE014-18
					diethyl ether ^5^	dietE014-18
					*n*-hexane (sox.) ^5^	hE014-18
					ethanol	etE014-18
*Combretum molle* R.Br. ex G.Don	Combretaceae	Ndagi	stem bark	AG191 (Makerere University herbarium, Uganda)	ethyl acetate ^2^	eE015
					ethanol ^2^	etE015
*Plectranthus hadiensis* (Forssk.) Schweinf. ex Sprenger	Lamiaceae	Kibwankulata	leaves	AG210 (Makerere University herbarium, Uganda)	diethyl ether ^4^	dietE016
					*n*-hexane ^4^	hE016

* Specimens have been digitized and are available for viewing at http://sernecportal.org/portal/ (accessed on 30 December 2017); ^1^ collected in Apr. 2016; ^2^ collected in Oct. 2015; ^3^ collected in Sep. 2013; ^4^ collected in Sep. 2016; ^5^ collected in Dec. 2017; sox. = Soxhlet extraction; sox. succ. = successive Soxhlet extraction.

**Table 2:** Results of COX-2 and COX-1 inhibition by medicinal plant samples from the Greater Mpigi region in Uganda; extracts are sorted from highest to lowest COX-2 sensitivity; IC_50_ values are given in µg/mL (positive control: ng/mL); SEM = standard error of the mean.

Extract ID	Plant Species	Type of Extract	IC_50_ ± SEM		Ratio COX-2 COX-1
			COX-2	COX-1	
eE005	*Leucas calostachys*	ethyl acetate	0.66 ± 0.66	7.76 ± 1.58	0.1
eE006	*Solanum aculeastrum*	ethyl acetate	1.74 ± 0.28	9.72 ± 0.28	0.2
hE006	*Solanum aculeastrum*	*n*-hexane	3.19 ± 0.43	3.99 ± 3.92	0.8
hE004	*Sesamum calycinum* subsp. *angustifolium*	*n*-hexane	3.65 ± 0.56	8.57 ± 2.03	0.4
dietE016	*Plectranthus hadiensis*	diethyl ether	4.55 ± 0.76	5.83 ± 3.79	0.8
eE004	*Sesamum calycinum* subsp. *angustifolium*	ethyl acetate	6.05 ± 0.20	11.47 ± 2.89	0.5
dietE014	*Warburgia ugandensis*	diethyl ether	13.33 ± 4.36	11.05 ± 1.43	1.2
etE009	*Zanthoxylum chalybeum*	ethnanol	16.07 ± 2.29	24.89 ± 4.16	0.7
dietE012	*Morella kandtiana*	diethyl ether	17.24 ± 2.79	15.01 ± 1.14	1.2
positive control	DuP-769	- (pure compound)	0.93 ± 0.20	>100.0	>0.001

**Table 3:** Resazurin bioassay growth inhibition results of medicinal plants from the Greater Mpigi region; MIC values with standard deviations are expressed as concentration (μg/mL). The maximum concentration at which extracts were tested was 500 μg/mL. Dashes indicate that a sample was not tested.

Scientific Name	Extract ID	*S. aureus* ATCC 25923	*E. coli* K12 ATCC 23716	*L. innocua* ATCC 33090	*L. monocytogenes* ATCC 15313
*Securidaca longipedunculata*	eE001	104.17 ± 29.46	>500	500.00 ± 0	-
	wE001	>500	>500	>500	-
	hE001	125.00 ± 0	>500	250.00 ± 0	250.00 ± 0
	mE001	83.33 ± 29.46	>500	>500	-
	smE001	104.17 ± 29.46	>500	>500	-
*Microgramma lycopodioides*	eE002	500.00 ± 0	>500	>500	-
	wE002	>500	>500	>500	-
	hE002	-	>500	>500	-
	mE002	250.00 ± 0	500.00 ± 0	500.00 ± 0	250.00 ± 0
	smE002	26.04 ± 7.37	>500	>500	-
*Ficus saussureana*	eE003	500 ± 0	500.00 ± 0	>500	-
	wE003	>500	>500	>500	-
	hE003	500.00 ± 0	500.00 ± 0	>500	-
	mE003	20.83 ± 7.37	500.00 ± 0	>500	-
	smE003	500 ± 0	500.00 ± 0	>500	-
*Sesamum calycinum* subsp. *angustifolium*	eE004	125.00 ± 0	500.00 ± 0	>500	-
	wE004	500 ± 0	>500	>500	-
	hE004	125.00 ± 0	500.00 ± 0	>500	-
	mE004	250.00 ± 0	500.00 ± 0	>500	-
	smE004	250.00 ± 0	>500	>500	-
	eE004-18	250.00 ± 0	-	-	-
	hE004-18	31.25 ± 0	-	-	-
*Leucas calostachys*	eE005	500.00 ± 0	>500	>500	-
	wE005	-	>500	>500	-
	hE005	62.50 ± 0	500.00 ± 0	>500	-
	smE005	500 ± 0	>500	>500	-
	eE005-18	500.00 ± 0	-	-	-
	hE005-18	104.17 ± 29.46	-	-	-
	mE005-18	500 ± 0	-	-	-
	smE005-18	104.17 ± 29.46	-	-	-
*Solanum aculeastrum*	eE006	500.00 ± 0	500.00 ± 0	>500	-
	wE006	500.00 ± 0	>500	>500	-
	hE006	125.00 ± 0	>500	>500	-
	smE006	11.72 ± 5.52	>500	>500	-
*Albizia coriaria*	eE007	250.00 ± 0	250.00 ± 0	>500	-
	etE007	500.00 ± 0	500.00 ± 0	>500	-
*Erythrina abyssinica*	eE008	83.33 ± 29.46	500.00 ± 0	>500	-
	etE008	62.50 ± 0	>500	>500	-
*Zanthoxylum chalybeum*	eE009	31.25 ± 0	-	>500	-
	etE009	500.00 ± 0	>500	>500	-
	etE017	500.00 ± 0	>500	>500	-
	dietE017	250.00 ± 0	>500	>500	-
	etE017a	>500	250.00 ± 0	-	-
	dietE017a	13.02 ± 3.62	>500	-	-
*Toddalia asiatica*	eE010	31.25 ± 0	>500	>500	-
	etE010	31.25 ± 0	>500	>500	-
	dietE010	20.83 ± 7.37	>500	>500	-
	etE010a	83.33 ± 29.46	>500	>500	-
*Harungana madagascariensis*	eE011	125.00 ± 0	250.00 ± 0	500.00 ± 0	>500
	etE011	57.29 ± 7.37	500.00 ± 0	500.00 ± 0	>500
	dietE011	13.02 ± 3.68	500.00 ± 0	41.67 ± 14.73	125 ± 0
	etE011a	125.00 ± 0	250.00 ± 0	500.00 ± 0	>500
	eE011-18	52.08 ± 14.73	-	-	-
	hE011-18	31.25 ± 0	-	-	-
	etE011-18	31.25 ± 0	-	-	-
	dietE011-18	52.08 ± 14.73	-	-	-
*Morella kandtiana*	eE012	250.00 ± 0	-	-	-
	etE012	500.00 ± 0	500.00 ± 0	>500	-
	etE012a	500.00 ± 0	250.00 ± 0	>500	-
	dietE012	250.00 ± 0	500.00 ± 0	>500	-
	eE012-18	500.00 ± 0	-	-	-
	dietE012-18	500.00 ± 0	-	-	-
*Cassine buchananii*	eE013	500.00 ± 0	500.00 ± 0	>500	-
	etE013	500.00 ± 0	500.00 ± 0	>500	-
	etE013a	500.00 ± 0	500.00 ± 0	>500	-
*Warburgia ugandensis*	etE014a	500.00 ± 0	500.00 ± 0	>500	-
	dietE014	31.25 ± 0	500.00 ± 0	500.00 ± 0	125 ± 0
	eE014-18	31.25 ± 0	-	-	-
	wE014-18	>500	-	-	-
	dietE014-18	31.25 ± 0	-	-	-
	hE014-18	31.25 ± 0	-	-	-
	etE014-18	41.67 ± 14.73	-	-	-
*Combretum molle*	eE015	500.00 ± 0	250.00 ± 0	>500	-
	etE015	500.00 ± 0	500.00 ± 0	>500	-
*Plectranthus hadiensis*	dietE016	104.17 ± 29.46	>500	>500	-
	hE016	62.50 ± 0	>500	>500	-
ciprofloxacin	-	0.19 ± 0.06	7.81 ± 0	0.12 ± 0.00	0.12 ± 0.00

## Data Availability

The data presented in this study are available in this article and in the Supplementary Material. Ethnobotanical data are available in Schultz, F.; Anywar, G.; Wack, B.; Quave, C.L.; Garbe, L.-A., Ethnobotanical study of selected medicinal plants traditionally used in the rural Greater Mpigi region of Uganda. *J. Ethnopharmacol.* 2020, 256, 112742.

## Supplementary Materials

The following are available online at https://www.mdpi.com/2223-7747/10/2/351/s1, Table S1: Results of the initial COX-2 extract library screen at 50 µg/mL, Table S2: Detailed data of the TPC determination and the DPPH assay, Table S3: Information on bacterial strains used in the study, Table S4: Procedure of the COX reaction step of the COX inhibition library screening assay, Figure S1: Plate layout and setup during the resazurin bioassay, Figure S2: Schematic description of the resazurin bioassay for growth inhibition, and references cited in the supplementary files.
