# Supplementary material for: Antiinflammatory Medicinal Plants from the Ugandan Greater Mpigi Region Act as Potent Inhibitors in the COX-2/PGH2 Pathway
Source: Plants (Basel). 2021 Feb 12;10(2):351. doi: 10.3390/plants10020351 (PMC7918315; doi:10.3390/plants10020351)
Supplement: Supplementary file 1 [file plants-10-00351-s001.pdf]

Antiinflammatory Medicinal Plants from the Ugandan Greater Mpigi Region act as potent inhibitors in the COX-2 / PGH_2 pathway

Fabien Schultz[1,2,*], Ogechi Favour Osuji[2], Barbara Wack[2], Godwin Anywar[3], and Leif-Alexander Garbe[2,4]

[1] Institute of Biotechnology, Faculty III - Process Sciences, Technical University of Berlin, Gustav-Meyer-Allee 25, Berlin, 13355, Germany
[2] Department of Agriculture and Food Sciences, Neubrandenburg University of Applied Sciences, Brodaer Str. 2, Neubrandenburg, 17033, Germany
[3] Department of Plant Sciences, Microbiology and Biotechnology, Makerere University, P.O Box 7062, Kampala, Uganda
[4] ZELT - Neubrandenburg Center for Nutrition and Food Technology gGmbH, Seestraße 7A, Neubrandenburg, 17033, Germany

* Correspondence: Fabien.Schultz@mailbox.tu-berlin.de; Tel.: +49-395-5693-2704

Supplementary information

Table of contents

Supplementary Table S1:
Results of the initial COX-2 extract library screen at 50 µg/mL page 3

Supplementary Table S2:
Detailed data of the TPC determination and the DPPH assay page 5

Supplementary Table S3:
Information on bacterial strains used in the study page 7

Supplementary Table S4:
Procedure of the COX reaction step of the COX inhibition library screening assay page 8

Supplementary Figure S1:
Plate layout and setup during the resazurin bioassay page 8

Supplementary Figure S2:
Schematic description of the resazurin bioassay for growth inhibition page 9

References cited in supplementary files page 9

Supplementary Table S1:
Results of the initial COX-2 extract library screen at 50 µg/mL

scientific name	extract ID	COX-2 %I at 50 µg/mL			
		0	1-40	41-80	>80
Securidaca longipedunculata	eE001	+	-	-	-
	smE001	+	-	-	-
	wE001	+	-	-	-
	mE001	+	-	-	-
	hE001	-	+	-	-
Microgramma lycopodioides	hE002	+	-	-	-
	mE002	-	+	-	-
	wE002	-	-	+	-
	smE002	+	-	-	-
	eE002	+	-	-	-
Ficus saussureana	smE003	-	-	+	-
	wE003	+	-	-	-
	eE003	-	+	-	-
	mE003	-	+	-	-
	hE003	+	-	-	-
Sesamum calycinum subsp. *angustifolium*	smE004	-	-	+	-
	mE004	+	-	-	-
	hE004	-	-	-	+
	hE004-18	nt			
	eE004	-	-	-	+
	eE004-18	nt			
	wE004	-	-	+	-
Leucas calostachys	eE005	-	-	-	+
	eE005-18	nt			
	smE005	-	-	+	-
	smE005-18	nt			
	wE005	-	+	-	-
	mE005-18	nt			
	hE005	-	-	+	-
	hE005-18	nt			
Solanum aculeastrum	eE006	-	-	-	+
	hE006	-	-	-	+
	wE006	-	+	-	-
	smE006	-	+	-	-
Albizia coriaria	etE007	+	-	-	-
	eE007	-	+	-	-
Erythrina abyssinica	etE008	-	+	-	-

	eE008	-	+	-	-
Zanthoxylum chalybeum	etE009	-	-	-	+
	eE009	-	-	+	-
	etE017	-	-	+	-
	etE017a	+	-	-	-
	dietE017	-	-	+	-
	dietE017a	+	-	-	-
Toddalia asiatica	etE010	-	+	-	-
	etE010a	-	+	-	-
	eE010	-	+	-	-
	dietE010	-	-	+	-
Harungana madagascariensis	etE011	-	-	+	-
	etE011a	-	+	-	-
	etE011-18	nt			
	eE011	+	-	-	-
	eE011-18	nt			
	dietE011	+	-	-	-
	dietE011-18	nt			
	hE011-18	nt			
Morella kandtiana	etE012	-	-	+	-
	etE012a	-	-	+	-
	eE012-18	nt			
	wE012-18	nt			
	dietE012	-	-	-	+
	dietE012-18	nt			
Cassine buchananii	etE013	+	-	-	-
	etE013a	-	+	-	-
	eE013	+	-	-	-
Warburgia ugandensis	dietE014	-	-	-	+
	dietE014-18	nt			
	eE014-18	nt			
	wE014-18	nt			
	hE014-18	nt			
	etE014a	-	-	+	-
	etE014-18	nt			
Combretum molle	etE015	+	-	-	-
	eE015	+	-	-	-
Plectranthus hadiensis	hE016	-	-	+	-
	dietE016	-	-	-	+

"nt" indicates that a sample was not tested.

Supplementary Table S2:
Detailed data of the TPC determination and the DPPH assay

Plant species and extract ID	TPC		DPPH scavenging activity	
	mg chlorogenic acid equivalent / g extract	SEM	EC50 (µg/ml)	SEM
Securidaca longipedunculata (eE001)	26.00	0.25	160.50	30.94
Securidaca longipedunculata (hE001)	24.56	0.06	68.10	26.36
Securidaca longipedunculata (mE001)	24.95	0.50	81.14	12.67
Securidaca longipedunculata (smE001)	24.92	0.40	55.31	9.45
Microgramma lycopodioides (eE002)	26.57	2.84	n.a. / no correlation	-
Microgramma lycopodioides (wE002)	5.02	0.53	n.a. / no correlation	-
Microgramma lycopodioides (hE002)	23.86	0.32	n.a. / no correlation	-
Microgramma lycopodioides (mE002)	23.95	0.63	91.27	23.09
Microgramma lycopodioides (smE002)	23.30	0.37	161.80	22.86
Ficus saussureana (eE003)	25.52	0.21	53.71	14.09
Ficus saussureana (wE003)	27.19	0.74	n.a. / no correlation	-
Ficus saussureana (hE003)	1.01	0.91	n.a. / no correlation	-
Ficus saussureana (mE003)	15.52	7.17	33.55	4.01
Ficus saussureana (smE003)	26.36	1.91	15.81	2.00
Sesamum calycinum subsp. *angustifolium* (eE004)	26.64	0.29	96.65	18.53
Sesamum calycinum subsp. *angustifolium* (wE004)	26.34	0.39	n.a. / no correlation	-
Sesamum calycinum subsp. *angustifolium* (hE004)	8.89	0.70	121.00	38.33
Sesamum calycinum subsp. *angustifolium* (mE004)	25.64	0.92	26.99	4.39
Sesamum calycinum subsp. *angustifolium* (smE004)	26.64	0.59	25.27	3.25
Leucas calostachys (eE005)	20.49	0.44	n.a. / no correlation	-
Leucas calostachys (wE005)	25.77	0.60	n.a. / no correlation	-
Leucas calostachys (hE005)	9.51	0.44	n.a. / no correlation	-
Leucas calostachys (smE005)	26.50	0.68	19.70	4.63
Solanum aculeastrum (eE006)	5.06	0.26	n.a. / no correlation	-
Solanum aculeastrum (wE006)	24.77	0.60	n.a. / no correlation	-
Solanum aculeastrum (hE006)	0.61	0.43	n.a. / no correlation	-
Solanum aculeastrum (smE006)	25.16	0.86	n.a. / no correlation	-
Albizia coriaria (eE007)	28.37	0.34	18.39	2.23

Albizia coriaria (etE007)	28.36	0.97	22.98	2.47
Erythrina abyssinica (eE008)	28.37	0.34	45.57	7.21
Erythrina abyssinica (etE008)	28.36	0.97	68.72	9.00
Zanthoxylum chalybeum (eE009)	32.39	0.23	106.00	25.33
Zanthoxylum chalybeum (etE009)	32.39	0.23	52.02	9.11
Zanthoxylum chalybeum (etE017)	26.39	0.24	n.a. / no correlation	-
Zanthoxylum chalybeum (etE017a)	28.19	0.13	44.60	7.12
Zanthoxylum chalybeum (dietE017)	23.81	0.82	91.61	31.57
Zanthoxylum chalybeum (dietE017a)	27.47	0.21	45.58	4.36
Toddalia asiatica (eE010)	3.00	0.29	n.a. / no correlation	-
Toddalia asiatica (etE010)	25.34	0.50	139.40	31.40
Toddalia asiatica (etE010a)	25.49	0.53	165.40	28.24
Toddalia asiatica (dietE010)	26.89	0.77	60.10	21.78
Harungana madagascariensis (eE011)	25.54	0.77	20.14	3.00
Harungana madagascariensis (etE011)	23.97	0.15	27.64	4.05
Harungana madagascariensis (etE011a)	32.09	0.45	33.19	4.35
Harungana madagascariensis (dietE011)	27.25	0.36	47.87	7.87
Morella kandtiana (etE012)	29.86	0.15	9.03	0.75
Morella kandtiana (etE012a)	29.88	0.48	8.97	8.97
Morella kandtiana (dietE012)	26.16	0.38	28.05	5.70
Cassine buchananii (eE013)	26.44	0.11	26.91	4.91
Cassine buchananii (etE013)	32.69	0.81	50.52	3.71
Cassine buchananii (etE013a)	26.75	0.78	23.78	2.39
Warburgia ugandensis (etE014a)	27.12	0.90	10.33	0.61
Combretum molle (eE015)	28.92	0.72	8.26	0.58
Combretum molle (etE015)	30.40	0.60	8.73	1.10
Plectranthus hadiensis (dietE016)	17.74	0.14	181.00	24.22
Plectranthus hadiensis (hE016)	8.03	0.38	23.63	12.71
quercetin	306.80	9.77	0.41	0.03
DMSO	0.00	0.00	-	-

Supplementary Table S3:
Information on bacterial strains used in the study

Species	Strain IDs	Characteristics*	Ref.
Escherichia coli	ATCC 23716<br>DSM # 498	Resistance: BAC, CLI, LIN, LZD, NYT, OXA, PEN-G, Q-D, TEC, VAN<br>K12 strain, mesophilic, rod-shaped, coliform, Gram-negative bacterium<br>Source: DSMZ	[1]
Listeria innocua	ATCC 33090<br>DSM # 20649	Resistance: no resistances reported<br>Isolate (bovine brain), mesophilic, rod-shaped, Gram-positive bacterium<br>Source: DSMZ	[2]
Listeria monocytogenes	ATCC 15313<br>DSM # 20600	Resistance: CST, NYT, PA<br>Intermediate resistance: PMB<br>Isolate (rabbit), mesophilic, rod-shaped, Gram-positive human pathogen<br>Source: DSMZ	[3]
Staphylococcus aureus	ATCC 25923<br>DSM # 1104	Resistance: ATM, CST, NYT<br>Intermediate resistance: PA<br>Human clinical isolate, mesophilic, Gram-positive human pathogen<br>Source: DSMZ	[4]

***abbreviations:**
ATM: aztreonam; BAC: bacitracin; CLI: clindamycin; CST: colistin; DSMZ: German Collection of Microorganisms and Cell Cultures GmbH; LIN: lincomycin; LZD: linezolid; NYT: nystatin; OXA: oxacillin; PA: pipemidic acid; PEN-G: penicillin G; PMB: polymyxin B; Q-D: quinupristin-dalfopristin (Synercid); TEC: teicoplanin; VAN: vancomycin

Supplementary Table S4:
Procedure of the COX reaction step of the COX inhibition library screening assay

	**Background tubes (2)**	**COX 100% initial activity tubes (2)**	**Sample (positive control) tubes**
**COX solution (heat inactivated)**	10 µL	-	-
**COX solution**	-	10 µL	10 µL
**COX buffer**	160 µL	160 µL	160 µL
**Heme solution**	10 µL	10 µL	10 µL
**Plant extract 10 mg/mL DMSO (or positive control)**	-	-	10 µL
**DMSO (sample vehicle)**	10 µL	10 µL	-
Incubate for 10 minutes at 37 °C			
**Arachidonic acid solution**	10 µL	10 µL	10 µL
Incubate for **exactly** 2.00 minutes at 37 °C			
**Saturated stannous chloride solution**	30 µL	30 µL	30 µL

Supplementary Figure S1:

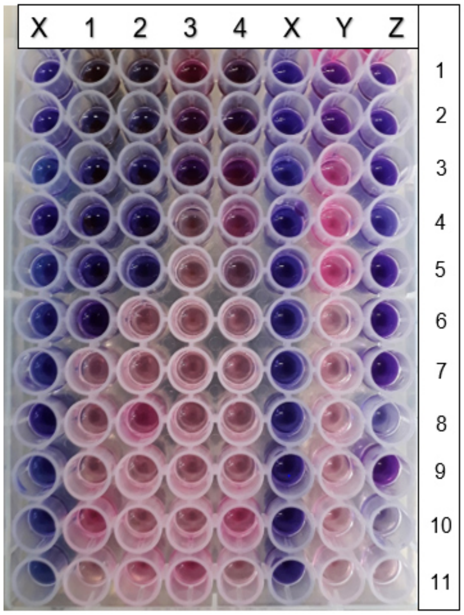

1

Figure S1. Plate layout and assay setup during the resazurin bioassay; violet wells indicate inhibition of cell viability; pink wells indicate bacterial growth; X: sterility control; Y: growth control; Z: positive control; 1-4: extract/sample solutions

Supplementary Figure S2:

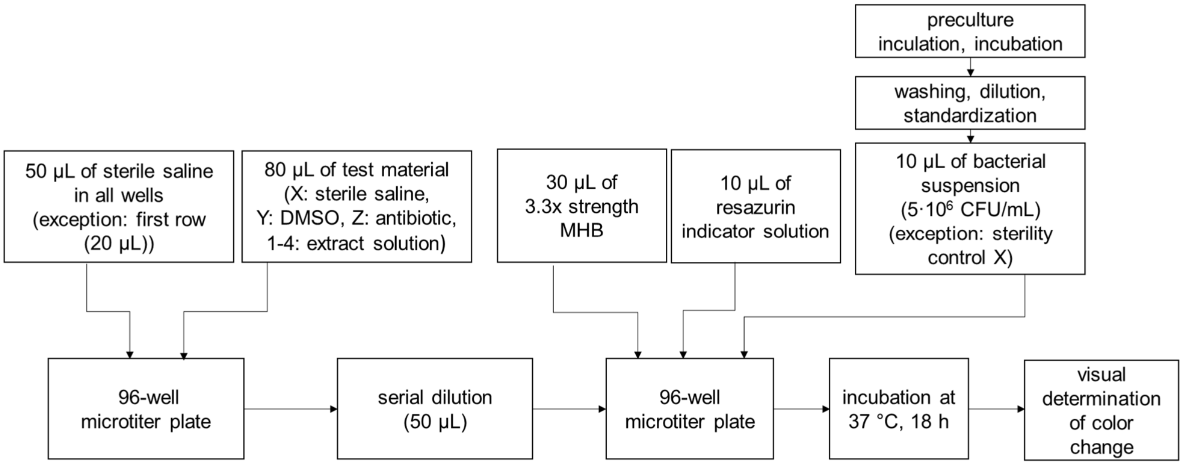

Figure S2: Schematic description of the resazurin bioassay for growth inhibition

References cited in supplementary files

1 BacDive. *E. coli K12 BacDive ID: 4414*, <https://bacdive.dsmz.de/strain/4414> (2020).

2 BacDive. *Listeria innocua 58 BacDive ID: 6871*, <https://bacdive.dsmz.de/strain/6871> (2020).

3 BacDive. *Listeria monocytogenes 53 XXIII BacDive ID: 6875*, <https://bacdive.dsmz.de/strain/6875> (2020).

4 BacDive. *Staphylococcus aureus Seattle 1945 BacDive ID: 14448*, <https://bacdive.dsmz.de/strain/14448 > (2020).
